# Validity and Reliability of the Self-administered Psycho-TherApy-SystemS (SELFPASS) Item Pool for the Daily Mood Tracking of Depressive Symptoms: Cross-sectional Web-Based Survey

**DOI:** 10.2196/29615

**Published:** 2021-10-18

**Authors:** Gwendolyn Mayer, Svenja Hummel, Nadine Gronewold, Neele Oetjen, Thomas Hilbel, Jobst-Hendrik Schultz

**Affiliations:** 1 Department of General Internal Medicine and Psychosomatics University Hospital Heidelberg Heidelberg Germany; 2 Electrical Engineering and Applied Sciences Westphalian University of Applied Sciences Gelsenkirchen Germany

**Keywords:** self-management, mood tracking, validity, reliability, item pool, questionnaire, depression, anxiety, mood assessment

## Abstract

**Background:**

e-Mental health apps targeting depression have gained increased attention in mental health care. Daily self-assessment is an essential part of e-mental health apps. The Self-administered Psycho-TherApy-SystemS (SELFPASS) app is a self-management app to manage depressive and comorbid anxiety symptoms of patients with a depression diagnosis. A self-developed item pool with 40 depression items and 12 anxiety items is included to provide symptom-specific suggestions for interventions. However, the psychometric properties of the item pool have not yet been evaluated.

**Objective:**

The aim of this study is to investigate the validity and reliability of the SELFPASS item pool.

**Methods:**

A weblink with the SELFPASS item pool and validated mood assessment scales was distributed to healthy subjects and patients who had received a diagnosis of a depressive disorder within the last year. Two scores were derived from the SELFPASS item pool: SELFPASS depression (SP-D) and SELFPASS anxiety (SP-A). Reliability was examined using Cronbach α. Construct validity was assessed through Pearson correlations with the Patient Health Questionnaire-9 (PHQ-9), the General Anxiety Disorder Scale-7 (GAD-7), and the WHO-5-Wellbeing-Scale (WHO-5). Logistic regression analysis was performed as an indicator for concurrent criterion validity of SP-D and SP-A. Factor analysis was performed to provide information about the underlying factor structure of the item pool. Item-scale correlations were calculated in order to determine item quality.

**Results:**

A total of 284 participants were included, with 192 (67.6%) healthy subjects and 92 (32.4%) patients. Cronbach α was set to .94 for SP-D and α=.88 for SP-A. We found significant positive correlations between SP-D and PHQ-9 scores (*r*=0.87; *P*<.001) and between SP-A and GAD-7 scores (*r*=0.80; *P*<.001), and negative correlations between SP-D and WHO-5 scores (*r*=–0.80; *P*<.001) and between SP-A and WHO-5 scores (*r*=–0.69; *P*<.001). Increasing scores of SP-D and SP-A led to increased odds of belonging to the patient group (SP-D: odds ratio 1.03, 95% CI 1.01-1.05; *P*<.001; SP-A: 1.05, 1.05-1.01; *P*=.01). The item pool yielded 2 factors: one that consisted of mood-related items and another with somatic-related items.

**Conclusions:**

The SELFPASS item pool showed good psychometric properties in terms of reliability, construct, and criterion validity. The item pool is an appropriate source for daily mood tracking in future e-mental health apps among patients with depression. Our study provides general recommendations for future developments as well as recommendations within the item pool.

## Introduction

### Mood Tracking and Symptom Monitoring in e-Mental Health Apps

e-Mental health apps targeting depression and anxiety play an increasing role in mental health care ranging from smartphone-based monitoring apps to extensive treatment applications [1-3]. Their evidence is regularly reviewed and shows that especially guided interventions are as successful as treatment-as-usual controls [4,5]. However, some barriers are still prevalent to fully exploit the potential of e-mental health apps in clinical practice. This might be owing to the fact that apps from clinical studies are not necessarily available in app stores, and choosing the right app poses difficulties among potential users [6]. Moreover, web-based interventions are subject to high attrition rates, as users may drop out soon after downloading apps [7,8]. The reasons for this low user engagement were identified beside others in the poor usability, the lack of user-centric design, and in their low ability to identify a crisis [9].

Regular mood tracking and symptom monitoring is a necessary step to identify sudden fluctuations that may hint at a suicidal crisis. The purpose of mood tracking in e-mental health apps for depression lies in the enhanced opportunities for self-reflection on mood and on their patterns and potential triggers of symptom aggravation [10]. Mood tracking is already implemented in a major share of e-mental health apps for depression. A recent review of Qu et al [11] on the functionality of 29 top-rated depression apps found that 19 (66%) included tools for mood tracking based on regular self-reports. Another review that focused on both, mobile apps and browser-based programs for depression, found mood-tracking functionalities in 86% [1]. Repeated measurement of a person’s mood or behavior in real time is referred to as ecological momentary assessment [12], and recent studies have shown the predictive power of mobile technology for depressive symptoms by capturing step counts and sedentary behavior [13], or vital parameters including sleep quality and heart rate [14]. However, currently available apps and programs hardly exploit the full technological potential of connecting wearable functionalities of the mobile phone or sensor data to regular mood queries [15].

Regarding anxiety apps, there is only scarce data on the availability and technical realization of mood tracking thus far. The last comprehensive study [2] that contributed to this question was published in 2017 and found 52 apps, 29% of which provided “emotional ratings.” It remains unclear if these ratings target mood tracking or rather initial screenings.

### Digital Mood-Related Self-assessment Tools

Digital versions of self-report scales regarding psychiatric symptoms show a comparable reliability to paper-pencil versions [16,17]. However, a detailed review of the diagnostic provenance of questions used in e-mental health apps has been missing thus far. The most frequently used questionnaire in depression management apps and chatbots is either the depression module of the Patient Health Questionnaire (PHQ-9) or self-developed questions [11,18].

The PHQ-9 is a brief depression screening instrument that has been validated across a variety of medical conditions [19,20]. The patients’ task is to self-assess the severity of 9 depressive symptom criteria over a span of the past 2 weeks; cut-off scores then allow for the assessment of depression severity. However, there are several disadvantages of the use of the PHQ-9 in a self-management app for depression. First, mood tracking in common e-mental health apps should be performed at least on a daily basis to provide timely information on the progression or worsening of symptoms and to make suggestions for symptom-specific interventions [1]. In such high-frequency self-assessment, it is important to ensure variety within the daily survey process to avoid the test routine. It has been shown that participants’ compliance to give valid responses to frequent mood assessment declines with high repetition rates [21]. The PHQ-9 is a self-assessment instrument that is not conceptualized for daily use, as the adherence to answering the same 9 questions every day may be low. Moreover, the questions refer to the past 2 weeks, and patients with depression may experience negative memory bias [22]. Finally, the PHQ-9 items screen depression criteria on the basis of DSM-IV [19], which differs slightly from the International Classification of Diseases (ICD) criteria. In general, DSM-IV diagnostic criteria require the presence of fewer symptoms than ICD-10 criteria, which reveals a slightly higher prevalence of depression in countries that rely on DSM rather than ICD [23]. Therefore, depression criteria that target an ICD-based diagnostic health system, similar to European countries, should not only rely on PHQ-9 items, even though it is an accepted tool in the clinical routine.

### Comorbidity of Depression and Anxiety

Recently since the introduction of the mixed anxiety-depressive disorder (F41.2) category in the ICD-10 in 1992, the complexity of differential diagnostics of anxiety and depression became evident [24]. Data on the prevalence of mental disorders in the United States reveal that 50%-60% of individuals with major depressive disorder (MDD) have also received a diagnosis of an anxiety disorder [25], and comparable data have been reported in Germany [26]. Theories on the relationship between the 2 diseases existed early on. Clark et al [27] described with their “tripartite model of anxiety and depression” that the general negative feelings are common to both syndromes, while the lack of positive feelings distinguishes depression from anxiety. According to them, only scales with symptom-specific content can sufficiently distinguish between the 2 syndromes [27,28]. In clinical practice, screening instruments are used specifically for 1 of the 2 diseases; for example, the PHQ-9 for depression [19] and the Generalized Anxiety Disorder Scale-7 (GAD-7) [29] are both modules of the PHQ [30]. However, some instruments cover both in the same test; for example, the Hospital Anxiety and Depression Scale (HADS) [31]. Developing an item pool for the usage of web-based daily mood assessments, while considering symptoms of comorbid anxiety in patients with depression, is necessary to provide symptom-specific intervention suggestions.

### Somatic Aspects in the Presentation of Depression

Somatic symptoms play an essential role in depressive symptomatology over the lifespan. Recent results have pointed out that depressive symptoms tend to shift with increasing age from a mood-related profile of symptoms to rather vegetative somatic symptoms including psychomotor agitation, gastrointestinal problems, or sleep disturbances [32]. Few vegetative symptoms are considered part of the diagnostic routine; for example, fatigue, loss of appetite, and sleep [33]. Understanding the individual burden profile has been found to be crucial for targeted treatment, as patients with higher values in self-criticism respond better to psychotherapy than those with a somatic symptom profile [34]. As a result, in developing an item pool for daily mood tracking in e-mental health apps, somatic symptoms should be considered to a certain extent. With regard to comorbid anxiety symptoms, somatic aspects of a panic disorder, which are associated with the characteristics of specific illnesses, have as well been detected early on [35].

### Scale Development Process

Scale development is a defined procedure that has been described comprehensively [36-38]. In short, 5 major steps are usually followed, beginning with (1) the generation of an item pool that is based on an extensive literature review and definition of the core concepts and the targeted population for the future scale. Item writing should involve simple and straightforward language while avoiding complex, ambiguous items. If a Likert-scale format is chosen, considerations about allowing midrange ratings are highly dependent on targets and core assumptions regarding the response behavior. Hence, no general conclusion regarding which response format is best is feasible [39]. (2) Qualitative analysis of the so far generated items and their content is the second step to (3) prepare a pilot with a small sample size. After that, (4) a larger evaluation study with a selected sample should be carried out by applying psychometric properties for reliability and different types of validity. Only then, norm values with a representative sample would be established [36]. The following investigation includes steps 1 to 4, while step 3 was included in a feasibility study that is currently being prepared for publication.

### Objectives

The aim of this study is the development of an item pool of mood-related questions for daily self-assessment in an e-mental health app to cover the main aspects of depressive symptoms in accordance with the diagnostic criteria of ICD-10 and comorbid symptoms of anxiety. We investigated the psychometric properties’ reliability, construct validity, criterion validity, and item-scale correlation of the item pool, which may be used for future self-management apps with suggestions for symptom-specific interventions. Thus, we aim to develop recommendations for the integration of mood-tracking items in future e-mental health apps.

## Methods

### The Self-administered Psycho-Therapy-SystemS App

The mobile app Self-administered Psycho-TherApy-SystemS (SELFPASS) was developed in a German study that received federal funding. This app was designed to improve the self-management of patients with depression on the basis of an individualized daily mood score. The target group comprises patients diagnosed with depression, who often wait a long time for a face-to-face psychotherapy [40]. The app allows for daily monitoring of depressive symptoms on the one hand and daily interventions to support patients on the other hand. SELFPASS does not claim to replace a face-to-face psychotherapy but rather to help patients during the waiting period, in order to bridge the treatment gap [41].

An item pool of 52 depression- and anxiety-related questions was developed to cover the main aspects of depressive symptoms and to provide suggestions for a pool of individualized interventions. Out of all items, 40 questions refer to depressive and 12 to anxiety symptoms. All questions are presented in Multimedia Appendix 1 in the original German version and translated to English (Multimedia Appendix 2). The item pool was developed jointly by mental health experts, who were part of an interdisciplinary team of psychologists and physicians in the clinic of psychosomatics. Items were translated by the authors and critically checked by active colleagues from the United States and other English-speaking countries. The main development approach was a rational construction strategy; that is, the process was guided mainly by theoretical considerations on the nature of depressive symptomatology [42]. The content of the depression items followed the major and minor symptoms of major depression in accordance with ICD-10 criteria, of which major symptoms are depressed mood, loss of interest, and loss of energy. Minor symptoms referred to the commonly listed ones including lack of concentration, feelings of worthlessness, guilt, pessimistic future expectations, suicidal ideation, sleep disturbances, and loss of appetite [33]. We used the following standardized diagnostic instruments as an additional source of information regarding the nature of symptom queries; however, all questions were rephrased: depression screening using 2 questions [43], PHQ-9 [19], HADS [31], and the short version of the Beck Depression Inventory (BDI-V) [44]. As these instruments are part of routine diagnostics [45] and are also validated in their German version [46,47], they provided an evidence-based foundation for the development of the SELFPASS items. We considered the following as anxiety symptoms: nervousness, excessive worry, accompanied by the inability to stop them, restlessness and not being able to relax, tendency to panic, and the fear of something awful happening. Anxiety questions were inclined toward clinical instruments (GAD-7), and further psychometric scales [48] including the State-Trait Anxiety Inventory (STAI) [49] and the anxiety subscale of the HADS, all of which have shown good validity in their German version [49,50]. Moreover, we included 2 of our own anxiety-related questions targeting the feeling of tightness in the chest and difficulty breathing, which are common symptoms of a panic attack [35].

All questions were rephrased. Each symptom was assessed through 4 different items in accordance with recommendations in the literature regarding test construction [51]: 2 of them were formulated in a negative direction and 2 in a positive direction to ensure diversion and to control for response bias (for example, “I have trouble concentrating on something” and “I can stick to one thing and concentrate fully on it”). The items were scored on a 6-point Likert scale ranging from 0=don’t agree to 5=agree. After starting the SELFPASS app, the user is asked to complete a daily self-assessment of at least 6 items from the SELFPASS item pool. Two of the questions relate to major depressive symptoms, 2 of them to minor symptoms, and the remaining 2 to anxiety symptoms. The number of daily questions may increase on the basis of the answers of the previous day, as the algorithm is designed to track individual symptoms. If a value of 3 is exceeded (positively formulated questions were automatically recoded by the algorithm), the symptom will be assessed again with an alternative formulation on the next day. Thus, the app is able to generate an individualized symptom profile based on the patient’s most prevalent current symptoms of the respective last 3 days and suggests 3 potentially appropriate interventions. For example, a result of highly prevalent anxiety symptoms will recommend relaxation interventions, while symptoms of ruminating and self-doubt will lead to an intervention suggestion for behavioral activation and cognitive restructuring.

### Study Design

We used a cross-sectional, web-based survey design to investigate the validity and reliability of the SELFPASS item pool. The study population consisted of 2 groups. The first group included healthy subjects, who reported not having any affective disorder within in the last 3 years. The second group included patients, who have received a diagnosis of any depressive disorder within the last year. We excluded patients with bipolar disorder, a psychosis, or suicidal ideation. Ethical approval for this study was granted by the Ethics Commission of the Medical Faculty of Heidelberg University (S-031/2020).

### Recruitment

The survey was made available on the internet via the soscisurvey.de [52] platform with 1 link each for patients and healthy subjects. The 2 versions included the same questionnaires except for demographic details. Thus, potentially psychiatric disorders in healthy subjects, who might take part in the study coincidentally, could be excluded. Exclusion criteria were a diagnosis with a bipolar affective disorder, a psychosis, or another psychiatric disorder within the last 3 years, and suicidality. Potentially suicidal participants were forwarded to an extra page with contact information for support. The group of healthy adults was recruited through social media channels and personal contact networks.

Patients were recruited within the Heidelberg University Hospital. They were contacted personally, via email or by post, and received a weblink to the study. Thus, the presence of a physicians’ diagnosis could be ensured, which is a prerequisite to assess criterion validity. Exclusion criteria were applied in advance.

### Validation Procedures

We provided 4 major parts of mood-related questions in random order: the German versions of the PHQ-9, GAD-7, WHO-5-Wellbeing-Scale (WHO-5) [53], as well as the SELFPASS item pool.

The PHQ-9 is a 9-item tool to assess depressive symptoms. The responses are rated on a 4-point Likert scale ranging from 0=not at all to 3=nearly every day. GAD-7 assesses 7 anxiety items on a scale from 0=not at all to 3=nearly every day. Both the PHQ-9 and GAD-7 consider a cut-off score of 10, with higher values indicating MDD [54] or moderate anxiety symptoms [55].

WHO-5 consists of 5 items, rated on a scale from 0=no time to 5=all of the time, for subjective well-being of the participant, with a high score indicating higher well-being. It was initially introduced by the World Health Organization (WHO) in 1998 as a first step in a 10-item screening process of depression and is usually followed by a diagnostic interview [56].

In this study, all psychometric instruments are used for construct validation, which refers to the alignment of the concepts measured by the instrument with their theoretical construct [57]. While the outcomes of the PHQ-9 and GAD-7 relative to SELFPASS scores serve as an indicator for convergent validity, the relationship between WHO-5 scores and SELFPASS scores demonstrate divergent validity. In turn, criterion validity targets the relationship between the results by the tested measures to an external criterion [57]. In our study, we considered the presence or absence of a valid physicians’ depression diagnosis as an external criterion to show the validity of the SELFPASS scores.

### Data Analysis

Statistical analysis was carried out in multiple steps using SPSS (version 24; IBM Corp) [58].

We excluded those data sets with a relative speed index of >1.75 as well as above 10% missing data per participant following pragmatic considerations and recommendations of the literature [59,60]. The option “no specification” for the SELFPASS items was treated as missing values for all statistical analyses.

After descriptive analysis of all participants (including means, SDs, and frequencies) we calculated subscales among the 40 SELFPASS depression (SP-D) and the 12 SELFPASS anxiety (SP-A) items.

We used Cronbach α to determine internal consistency of the subscales. Cronbach α>.80 was considered a threshold for acceptance [61,62].

Convergent validity was assessed using Pearson correlation coefficients of SP-D with PHQ-9 and SP-A with GAD-7. Pearson correlation coefficients were also computed for WHO-5 and SP-D and for the sum of SP-A and SP to assess discriminant validity. Following Cohen’s [63] definition, *r*>0.30 can be interpreted as a moderate and *r*>0.50 a strong correlation.

Logistic regression analysis was performed to analyze the power of SP-D and SP-A to distinguish between patients and healthy subjects. The results will serve as criterion validation. We considered the *R*^2^ value in accordance with Nagelkerke [64] as good if it fell within a range of *R^2^*=0.20 and 0.40.

We carried out an explorative factor analysis to investigate the underlying factor structure of the items. At first, we applied the analysis to the whole sample, and a more detailed investigation studied the factor structure within the data of only the patients. The Kaiser-Meyer-Olkin (KMO) measure of sampling adequacy was interpreted as acceptable if its value exceeded 0.50 [65]. The Bartlett test [66] of sphericity revealed a significant (*P*<.001) result, which indicated that the items were appropriate for factor analysis [67]. We chose a principal axis factor analysis approach followed by oblimin rotation, as recommended for factors that might show intercorrelations [67].

Finally, we correlated the items’ values with the respective scale value to obtain the individual item-scale correlation for the analysis of the discriminatory power of each item. We applied the rule of thumb to remove items with a correlation below *r*=0.30 [68,69]. The share of missing values of single items and their inter-item correlations was as well interpreted as an indicator for low item quality. We considered items with more than 20 inter-item correlations below *r*=0.20 as critical in accordance with recommendations from the literature [70]. However, the final decision about deletion of the respective items was made jointly together after the analysis of missing values and discriminatory power. All results on the level of single items are reported in Multimedia Appendix 3.

A level of *P*<.05 was considered significant in all statistical tests.

## Results

### Participants

In total, 329 participants responded to the web-based questionnaire from end-March to mid-August 2020. After excluding respondents with a relative speed index of >1.75 (n=31) and more than 10% of missing items (n=4), as well as healthy subjects with a psychiatric diagnosis (n=10), the final sample comprised 284 participants. The sample consisted of 192 (67.6%) healthy subjects and 92 (32.4%) patients. The demographic characteristics of the participants are shown in Table 1.

**Table 1:** Demographic characteristics of the study sample (N=284).

Characteristics			Participants				
			Healthy subjects (n=192)		Patients (n=92)		Total
Age (years), mean (SD)			29.02 (9.70)		40.46 (14.91)		32.73 (12.8)
**Gender, n (%)**							
	Male	43 (22.4)		38 (41.3)		81 (28.5)	
	Female	148 (77.1)		53 (57.6)		201 (70.8)	
	Other	1 (0.5)		1 (1.1)		2 (0.7)	
**Family status, n (%)**							
	Single	162 (84.4)		46 (50.0)		208 (73.2)	
	Married	19 (9.9)		31 (33.7)		50 (17.6)	
	Divorced	7 (3.6)		7 (7.6)		14 (4.9)	
	Widowed	0 (0)		2 (2.2)		2 (0.7)	
	Separated	0 (0)		5 (5.4)		5 (1.8)	
	Other	4 (2.1)		1 (1.1)		5 (1.8)	
**Level of education, n (%)**							
	No degree	0 (0)		3 (3.3)		3 (1.1)	
	High school	64 (33.3)		37 (40.2)		101 (35.6)	
	College	123 (64.1)		47 (51.1)		170 (59.9)	
	Dissertation/PhD	5 (2.6)		2 (2.2)		7 (2.5)	
	Other	0 (0)		3 (3.3)		3 (1.1)	
**Profession, n (%)**							
	Self-employed	3 (1.6)		1 (1.1)		4 (1.4)	
	Worker	5 (2.6)		9 (9.8)		14 (4.9)	
	Civil servant	3 (1.6)		3 (3.3)		6 (2.1)	
	Employee	51 (26.6)		37 (40.2)		88 (31.0)	
	Not working	3 (1.6)		11 (12.0)		14 (4.9)	
	Student/pupil	123 (64.1)		17 (18.5)		140 (49.3)	
	Retired	3 (1.6)		5 (5.4)		8 (2.8)	
	Other	1 (0.5)		9 (9.8)		10 (3.5)	

### Psychometric Scores and SELFPASS Subscales

The mean scores of all psychometric scales and the SELFPASS subscales are presented in Table 2. On average, the healthy subjects had lower scores on all scales than the patients, except for the WHO-5.

**Table 2:** Scores of healthy subjects and patients on the psychometric scales.

Scale	Healthy subjects (n=192), mean (SD)	Patients (n=92), mean (SD)
SELFPASS^a^ for depression	53.33 (24.62)	87.40 (29.27)
SELFPASS for anxiety	18.63 (9.65)	31.37 (11.62)
Overall Self-administered Psycho-TherApy-SystemS	71.96 (32.37)	118.77 (38.63)
Patient Health Questionnaire-9	5.39 (3.95)	10.40 (5.23)
General Anxiety Disorder Scale-7	5.03 (3.88)	8.76 (4.53)
WHO-5-Wellbeing-Scale	14.46 (4.86)	8.57 (5.47)

^a^ SELFPASS: Self-administered Psycho-TherApy-SystemS.

### Reliability

The internal consistency of the 40-item SP-D subscale and the 12-item SP-A subscale was assessed from a Cronbach α of .94 for SP-D (n=240) and .88 for SP-A (n=275).

### Construct Validity

Table 3 shows the results of construct validity analysis. Regarding convergent validity, the SELFPASS depression and anxiety subscale show positive correlations with the scores of PHQ-9 and GAD-7, respectively, as valid depression and anxiety measures. The data reveal significant negative correlations among all 3 SELFPASS scores with WHO-5 scores, showing discriminant validity.

**Table 3:** Pearson correlation analysis to determine the correlation between the scores of SELFPASS^a^ subscales with those of the Patient Health Questionnaire-9, General Anxiety Disorder Scale-7, and WHO-5-Wellbeing-Scale.

		Pearson correlation coefficient				
		SELFPASS for depression		SELFPASS for anxiety		Overall SELFPASS
**Convergent validity**						
	Patient Health Questionnaire-9	0.87^b^	0.74^b^			
	General Anxiety Disorder Scale-7	0.70^b^	0.80^b^			
**Discriminant validity**						
	WHO-5-Wellbeing-Scale	–0.80^b^	–0.69^b^		–0.80^b^	

^a^ SELFPASS: Self-administered Psycho-TherApy-SystemS.

^b^*P*<.01.

### Criterion Validity

The results of the logistic regression analysis for patients and healthy subjects are presented in Table 4. The overall model was significant (*χ*^2^_2_=91.39; *P*<.001; N=284) as well as the coefficients SP-D and SP-A. Increasing scores for depression and anxiety increase the odds of being part of the patient group. Details are presented in Table 4.

**Table 4:** Results of logistic regression analysis indicating the probability of being part of the patient group on the basis of the scores of the SELFPASS depression and anxiety subscales^a^.

Predictor	β (SE)	*P* value	Odds ratio (95% CI)
Constant	–4.15 (0.49)	<.001	0.02
SELFPASS for depression	0.03 (0.01)	<.001	1.03 (1.01-1.05)
SELFPASS for anxiety	0.05 (0.02)	.01	1.05 (1.01-1.10)

^a^SELFPASS: Self-administered Psycho-TherApy-SystemS; Omnibus test: *χ*^2^_2_=91.39; *P*<.001; Hosmer–Lemeshow test: *χ*^2^_8_=7.16; *P*=.52; Nagelkerke *R^2^*=0.38.

### Exploratory Factor Analysis and Item Analysis

A principal axis analysis with the whole sample revealed a 2-factor solution after scree plot analysis. The 2 factors accounted for 36.70% of the variance, and oblique rotation was performed. The intercorrelation of the 2 factors was *r*=0.40. The KMO measure of sampling adequacy was 0.94. Four items showed inter-item correlations with coefficients less than *r*=0.20 in more than 20 cases (SP 20, 23, 25, and 39). Item 25 was not answered in 23 of 240 (8.1%) cases. Item-scale correlations were calculated and helped evaluate the following items as critical: SP 20, 23, 24, 25, 33, and 41. After excluding them, another exploratory factor analysis explained 40.95% of the variance. Details on the level of single items as well as the item-scale correlations are presented in Multimedia Appendix 3.

The patient subgroup showed a KMO measure of 0.70. A principal axis analysis with the patient sample accounted for 32.77% of the variance. After excluding the critical items, 35.90% of the variance was accounted for. The intercorrelation of the 2 factors was *r*=0.21.

The items as well as the loadings of the items in the whole sample and the patient subsample are presented in Table 5.

**Table 5:** Items, associated symptoms, and results of the principal axis factor analysis of the whole sample after oblique rotation (N=284), and of the patients’ sample (n=92).

Item			Item formulation		Symptom		Factor 1 (all)		Factor 1 (patients)		Factor 2 (all)		Factor 2 (patients)
**SELFPASS^a^ for depression**													
	SP1	I feel depressed, sad or hopeless.		CS^b^1		0.73		0.73					
	SP2	I easily burst into tears.		CS1		0.40		0.42					
	SP3	I am cheerful and in good spirits.		CS1		0.87		0.82					
	SP4	I feel easy and carefree.		CS1		0.88		0.77					
	SP5	I have much less desire and enjoyment for things I usually like to do.		CS2		0.76		0.72					
	SP6	I have no interest in people around me.		CS2		0.33		0.37					
	SP7	I can laugh at funny moments.		CS2		0.54		0.54					
	SP8	I can enjoy pleasant things and be happy about them.		CS2		0.63		0.53					
	SP9	I feel exhausted and sluggish.		CS3		0.82		0.68					
	SP10	I can't force myself to do anything.		CS3		0.61		0.47					
	SP11	Decision making is easy for me.		CS3		0.48		0.34					
	SP12	I am full of drive and energy.		CS3		0.86		0.83					
	SP13	I have problems in concentrating on something.		AS^c^1		0.74		0.72					
	SP14	My thoughts keep on slipping away.		AS1		0.63		0.64					
	SP15	I can dwell on one thing with my full concentration.		AS1		0.69		0.59					
	SP16	I am not easily distracted.		AS1		0.54		0.49					
	SP17	I am just not good enough.		AS2		0.54		0.40					
	SP18	Others can do things much better than I can.		AS2		0.35		0.35					
	SP19	I am satisfied with myself.		AS2		0.76		0.60					
	SP20^d^	I take care of my appearance.		AS2		0.20						0.32	
	SP21	I should have done things much differently in the past.		AS3		0.43		0.40					
	SP22	I have made mistakes. It´s not surprising I feel bad.		AS3		0.47		0.51					
	SP23^d^	I am not perfect. But who is?		AS3		0.29		0.24					
	SP24^d^	I don’t deserve to feel bad.		AS3		-0.03		-0.23					
	SP25^d^	It can only get worse.		AS4		0.19		0.14		0.19			
	SP26	The future has nothing to offer for me.		AS4		0.53		0.62					
	SP27	I am looking forward to the future.		AS4		0.67		0.67					
	SP28	Time heals all wounds. Everything will be alright.		AS4		0.61		0.46					
	SP29	Sometimes I think it would be better to be dead.		AS5						0.59		0.45	
	SP30	I think a lot about death.		AS5				0.33		0.41			
	SP31	I think about putting hands on myself.		AS5						0.56		0.31	
	SP32	I have already thought about how to kill myself.		AS5						0.63		0.46	
	SP33^d^	I sleep too much.		AS6		0.16						0.35	
	SP34	I have trouble falling asleep and/or wake up constantly.		AS6		0.55		0.40					
	SP35	My sleep was restful and sufficient.		AS6		0.76						0.45	
	SP36	I slept well.		AS6		0.74		0.44					
	SP37	I feel a constant hunger or appetite for food.		AS7				-0.06		0.08			
	SP38	I don’t feel like eating anything.		AS7		0.33		0.30					
	SP39	I have a good appetite.		AS7		0.28						0.27	
	SP40	I eat enough and I follow a balanced diet.		AS7		0.42						0.43	
**SELFPASS for anxiety**													
	SP41^d^	I hope that I don’t get sick.		CA^e^				0.18		0.07		-0.36	
	SP42	Sometimes I have an oppressive feeling in my stomach.		CA		0.56		0.55					
	SP43	I am worried that something terrible will happen.		CA		0.37		0.59					
	SP44	Sometimes I start panicking suddenly.		CA		0.52		0.58					
	SP45	When I’m worried, I still can keep my control.		CA		0.56		0.46					
	SP46	Disturbing thoughts run through my mind.		CA		0.52		0.66					
	SP47	I’m calm.		CA		0.79		0.78					
	SP48	When I think of my current affairs, I get anxious.		CA		0.59		0.61					
	SP49	I feel safe and secure		CA		0.77		0.69					
	SP50	I’m worried about something going wrong soon.		CA				0.48		0.43			
	SP51	Sometimes I feel tightness in my chest.		CA		0.66		0.63					
	SP52	Sometimes I can’t breathe properly.		CA		0.53		0.59					
	Eigen value					16.95		14.27		5.65		4.10	

^a^SELFPASS: Self-administered Psycho-TherApy-SystemS.

^b^CS: core symptom.

^c^AS: additional symptom.

^d^items that should be excluded or reformulated.

^e^CA: comorbid anxiety.

## Discussion

### Principal Findings

This study aimed to investigate the psychometric properties of an item pool of mood-related questions for daily self-assessment, which cover symptoms of depression and comorbid anxiety to make suggestions for symptom-specific interventions. The item pool was developed for the use within the e-mental health app SELFPASS and for future developments. Through a web-based cross-sectional survey design, the instrument emerged as reliable and valid. The psychometric properties are shown in a representative study population of healthy subjects and patients with a diagnosis of depression within the past year. Considering that average PHQ-9 scores of 10.4 indicate a moderate severity of depression [54] and GAD-7 scores of 8.76 indicate mild symptoms of anxiety [55], the patients seemed to be considerably affected and were hence eligible to test the SELFPASS item pool appropriately.

Both subscales assessing symptoms of depression (SP-D) and anxiety (SP-A) showed high correlations with standardized psychometric instruments (PHQ-9 and GAD-7). This demonstrates a high inherent construct validity. The WHO-5 as an indicator of subjective well-being was negatively correlated with SP-D and SP-A, which in turn reveals good discriminatory construct validity. The negative association of the WHO-5 with depression and anxiety scales has already been shown in other validation studies [71,72]. Moreover, the results of the SELFPASS subscales in the whole sample were able to predict the affiliation to the patient group, which was interpreted as concurrent criterion validity. As expected, increasing scores for depression and anxiety measured through SELFPASS increase the odds of being part of the patient group.

An exploratory factor analysis indicated an underlying 2-factor structure of the item pool that covered a mood-related factor on the one hand and a somatic factor on the other hand in the whole sample. The factor structure of the patient sample even increased this structure, including more items in the second factor, which were related to paying attention to appearance, suicidal thoughts, sleep, appetite, and the fear of becoming sick. There was 1 item that did not fully seem to fit to this interpretation (“I’m worried about something going wrong soon”). A potential explanation might lie in the timing of investigation, as many patients were concerned with becoming infected with SARS-CoV-2 at that time [73].

A closer investigation of screening instruments for depression shows that validation studies of the PHQ-9 among different populations; for example, in palliative care, these instruments show a comparable 2-fold factor structure of 1 factor focusing on cognitive and affective aspects and another one relating to somatic symptoms [74]. Although for palliative patients with a high somatic burden, this might be an obvious result; however, similar results were obtained in a psychiatric sample [75]. Therefore, somatic aspects of depression might reveal an underlying structure of depression and anxiety, which is reminiscent of the “Tripartite model of depression and anxiety,” which states that beside the already described “generally negative affect” as a third factor “somatic symptoms” [27,75]. Moreover, suicidal ideation has been identified as particularly crucial among patients with depression with somatic syndrome [74,75].

Enhancing the quality of e-mental health apps, especially with regard to a successful crisis management in case of symptom exacerbation, has already been identified as a necessary step to reduce dropout rates and increase adherence to digital interventions [9]. The integration of appropriate mood-tracking items, as provided by the presented item pool in future e-mental health apps, is a necessary measure in integrating digital assistance in routine clinical practice.

### Recommendations for the Use of the SELFPASS Item Pool

The SELFPASS item pool is suitable for daily mood assessment in any kind of web-based intervention or e-mental health app. It is designed for highly frequent repetitive use providing approximately 6 items every day out of the total item pool based on the results of the previous days. Following this purpose, we recommend reformulating items 20, 23, 24, 25, 33, and 41 for appropriate use and future validation studies. For any other use, the items may be dropped as well. Although item 31, 32, 37, and 39 also showed a lack of quality after item analysis, we recommend retaining these items as they assess important depressive symptoms.

Based on our experience with the development and validation of the SELFPASS item pool, some general recommendations may be provided to ensure optimization of the items (Table 6). Simultaneously, we summarized recommending conclusions within the SELFPASS item pool.

**Table 6:** Recommendations for the future use of the SELFPASS^a^ item pool.

Topic	General recommendation	Recommendation for use within the SELFPASS item pool
Item presentation	Provide a diversion in item presentation to increase adherence.	Ask for 2 main symptoms, 2 additional symptoms and two anxiety symptoms per day. If 1 symptom exceeds a critical score, pursue this symptom with an alternative item.
Symptom coverage	Cover somatic, cognitive, and emotional aspects of symptomatology.	Provide a random choice of daily items following the rules described above to cover a broad range of symptoms.
Crisis management	Include crisis management in case of positive answers to suicidal ideation.	Same as the general recommendation.
Discriminatory power	Evaluate each question with regard to whether it might be able to sufficiently differentiate between healthy subjects and patients. Hence, symptoms including increased appetite and sleep were excluded by the Beck Depression Inventory [76], but we do not follow this approach.	Reformulate items 20, 23, 24, 25, 33, and 41. A closer focus on the manifestation of these symptoms in patients with depressive and anxiety symptoms is recommended.
External validation	Provide validation of single assessments, such as BMI, as matching self-assessments of body weight or sleep parameters delivered by a sensor as control for self-assessed sleep quality.	Same as the general recommendation.
Variation	Provide positive and negative directions of items.	Same as the general recommendation.
Targeted use	Be aware of the target population of the questionnaire.	The item pool addresses patients with a pre-existing diagnosis of depression for daily monitoring of their symptoms.

^a^SELFPASS: Self-administered Psycho-TherApy-SystemS.

### Directions of Future Research

With the introduction of the ICD-11, some diagnostic criteria for depressive episodes will change, which should be considered for further use of the SELFPASS item pool. It is expected that the previous 3 main symptoms of depression will be reduced to 2. Fatigue and lack of drive are then considered additional symptoms [76]. Feelings of worthlessness and guilt will be summarized to one, as well as sleep and appetite. Then, psychomotor agitation or retardation, formerly included in the somatic symptoms, will be considered as own symptoms [77]. This could be relevant for apps that provide symptom-specific intervention suggestions as SELFPASS does.

Moreover, much evidence has been provided that ecological momentary assessments delivered through mobile data have the potential to become behavioral markers for mental health symptoms; for example, movement profiles collected from GPS data and circadian sleep rhythms recorded through phone usage [78]. Unfortunately, mental health apps beyond scientific studies hardly make use of data processing technologies that allow for personalized intervention content based on the activities of the users [15]. Future studies may focus on validation approaches of different data sources in one instrument; for example, daily questions for self-assessment, sensor data, and further behavioral markers delivered through mobile devices. Validation with the help of sensor data, finally, is a powerful approach to verify the validity of the item pool during use over a longer period, to ascertain its test-retest validity. As of this writing, the item pool might serve as an ecological momentary assessment tool based on questions only, which might be asked several times per day.

Beside the somatic symptoms outlined above and considered in our item pool, it should be noted that depression is often accompanied by several somatic conditions including obesity, cardiovascular diseases, diabetes, pain, or even multimorbidity [79]. However, existing monitoring apps thus far do not target the simultaneous management of mental and somatic conditions, except for medication adherence [80]. Future approaches should build upon existing results and integrate single solutions to transdiagnostic apps that thus far exist within the field of mental health [81] but rather do not pertain to mental and physical conditions. Our item pool might serve as a valuable source and can be complemented by physical conditions.

### Limitations

There are some limitations to this study, which should be considered. First, the data were collected during the 2020 COVID-19 pandemic, which might have had an impact on the mental health of the participants [82,83]. In addition, the patient and healthy subject group differ in terms of group size, age, and gender. Regarding criterion validity, only a diagnosis of depression was defined as an inclusion criterion for the patient group. Owing to the described comorbidity of both disorders, anxiety was nevertheless also considered an external criterion to determine criterion validity. Another study should focus on comparing patient populations with validated diagnoses of both depression and anxiety separately. Thus, another confirmatory factor analysis should be performed to clarify the factor structure of the item pool. We provide a validation of a whole item pool of 52 questions, although the number of the daily questions is much lower based on the symptom profile of the respective user. A validation of this process of choice would require a substantially high number of patients with different symptomatology, which was not possible to carry out in our study design. Moreover, we did not carry out cognitive interviewing with a small sample size of patients, which is sometimes carried out during test construction [84]. However, as the symptoms of depression are already well studied, we decided to rely on the experience of the existing diagnostic instruments. As a final limitation, delusionary and psychotic symptoms that are relevant in case of a psychotic depression were not considered in the SELFPASS item pool. Psychotic depression is a subtype of depression in ICD-10, and no changes regarding ICD-11 are expected [85]. Thus, if future e-mental health apps focus as well on more severe forms of depressive disorders, further items should be added and validated.

### Conclusions

The SELFPASS item pool is a valid and reliable source for daily self-assessment in e-mental health apps. It follows diagnostic standards of depression and comorbid anxiety symptoms. The item pool is a valuable source of questions for daily mood tracking in future e-mental health apps. Further developments have to focus on optimizing the wording of single items as well as the adaptations that are expected from ICD-11 in the near future.

## Appendix

Multimedia Appendix 1 Self-administered Psycho-TherApy-SystemS (SELFPASS) question catalogue (German original).

Multimedia Appendix 2 Self-administered Psycho-TherApy-SystemS (SELFPASS) question catalogue (English translation).

Multimedia Appendix 3 Results of the item analysis of SELFPASS depression (SP-D) and SELFPASS anxiety (SP-A): Means, SD, Item-scale-correlation, missing values (N, %) and number significant inter-item correlations per item (N). SELFPASS: Self-administered Psycho-TherApy-SystemS.

## Abbreviations

BDI-V: Beck Depression Inventory
GAD-7: General Anxiety Disorder Scale-7
HADS: Hospital Anxiety and Depression Scale
ICD: International Classification of Diseases
KMO: Kaiser-Meyer-Olkin
PHQ-9: Patient Health Questionnaire-9
SELFPASS: Self-administered Psycho-TherApy-SystemS
SP-A: SELFPASS anxiety
SP-D: SELFPASS depression
STAI: State-Trait Anxiety Inventory
WHO: World Health Organization
WHO-5: WHO-5-Wellbeing-Scale
