# Supplementary material for: Validity and Reliability of the Self-administered Psycho-TherApy-SystemS (SELFPASS) Item Pool for the Daily Mood Tracking of Depressive Symptoms: Cross-sectional Web-Based Survey
Source: JMIR Ment Health. 2021 Oct 18;8(10):e29615. doi: 10.2196/29615 (PMC8561414; doi:10.2196/29615)
Supplement: Multimedia Appendix 1 [file mental_v8i10e29615_app1.docx]

| **No** | **Item^1^** | **To be recoded** |
| --- | --- | --- |
| 1 | Ich fühle mich niedergeschlagen, traurig, bedrückt oder hoffnungslos. |  |
| 2 | Ich breche leicht in Tränen aus. |  |
| 3 | Ich bin fröhlich und guter Dinge. | X |
| 4 | Ich fühle mich leicht und unbeschwert. | X |
| 5 | Ich habe deutlich weniger Lust und Freude an Dingen, die ich sonst gerne tue. |  |
| 6 | Die Menschen in meiner Umgebung interessieren mich nicht. |  |
| 7 | Ich kann über lustige Momente lachen. | X |
| 8 | Ich kann angenehme Dinge genießen und mich an ihnen freuen. | X |
| 9 | Ich fühle mich erschöpft und lustlos. |  |
| 10 | Ich kann mich zu keiner Tätigkeit durchringen. |  |
| 11 | Entscheidungen fallen mir leicht. | X |
| 12 | Ich bin voller Tatendrang und Energie. | X |
| 13 | Ich habe Probleme damit, mich auf etwas zu konzentrieren. |  |
| 14 | Meine Gedanken gleiten ständig ab. |  |
| 15 | Ich kann bei einer Sache bleiben und mich voll darauf konzentrieren. | X |
| 16 | Ich lasse mich nicht so leicht ablenken. | X |
| 17 | Ich bin einfach nicht gut genug. |  |
| 18 | Andere können Vieles besser als ich. |  |
| 19 | Ich bin mit mir zufrieden. | X |
| 20² | Ich achte auf mein Aussehen.^2^ | X |
| 21 | Ich hätte in der Vergangenheit Vieles anders machen müssen. |  |
| 22 | Ich habe Fehler gemacht. Kein Wunder, dass es mir schlecht geht. |  |
| 23² | Ich bin nicht perfekt. Aber wer ist das schon?^2^ | X |
| 24² | Ich habe es nicht verdient, dass es mir schlecht geht.^2^ | X |
| 25² | Es kann nur schlechter werden.^2^ |  |
| 26 | Die Zukunft hat mir nichts zu bieten. |  |
| 27 | Ich freue mich auf die Zukunft. | X |
| 28 | Die Zeit heilt alle Wunden. Es wird schon alles gut werden. | X |
| 29 | Manchmal denke ich, dass es besser wäre tot zu sein. |  |
| 30 | Ich denke viel über den Tod nach. |  |
| 31 | Ich denke darüber nach, mir etwas anzutun. |  |
| 32 | Ich habe mir schon Schritte überlegt, wie ich mein Leben beenden könnte. |  |
| 33² | Ich schlafe zu viel.^2^ |  |
| 34 | Ich schlafe schlecht ein und/oder wache ständig auf. |  |
| 35 | Mein Schlaf war erholsam und ausreichend. | X |
| 36 | Ich habe gut geschlafen. | X |
| 37 | Ich habe ständig Hunger oder Appetit. |  |
| 38 | Ich habe keine rechte Lust auf Essen. |  |
| 39 | Ich habe einen guten Appetit | X |
| 40 | Ich esse genug und ernähre mich ausgewogen. | X |
| 41² | Ich hoffe, dass ich nicht krank werde.^2^ |  |
| 42 | Ich habe manchmal ein beklemmendes Gefühl im Magen. |  |
| 43 | Ich habe Sorge, dass etwas Schreckliches passieren wird. |  |
| 44 | Manchmal bekomme ich aus heiterem Himmel Panik. |  |
| 45 | Wenn ich mir einmal Sorgen mache, behalte ich trotzdem die Kontrolle darüber. | X |
| 46 | Mir gehen beunruhigende Gedanken durch den Kopf. |  |
| 47 | Ich bin gelassen. | X |
| 48 | Wenn ich an meine derzeitigen Angelegenheiten denke, werde ich ganz unruhig. |  |
| 49 | Ich fühle mich sicher und geborgen. | X |
| 50 | Ich fürchte, dass schon bald etwas schief gehen wird. |  |
| 51 | Mich überkommt manchmal ein Gefühl von Enge in der Brust. |  |
| 52 | Manchmal kann ich gar nicht richtig atmen. |  |

1 Likert-Skala von 0 = stimme überhaupt nicht zu bis 5 = stimme voll zu

2 Items die ausgeschlossen und/oder umformuliert werden sollten
