# Supplementary material for: Validity and Reliability of the Self-administered Psycho-TherApy-SystemS (SELFPASS) Item Pool for the Daily Mood Tracking of Depressive Symptoms: Cross-sectional Web-Based Survey
Source: JMIR Ment Health. 2021 Oct 18;8(10):e29615. doi: 10.2196/29615 (PMC8561414; doi:10.2196/29615)
Supplement: Multimedia Appendix 2 [file mental_v8i10e29615_app2.docx]

| **No** | **Item^1^** | **To be recoded** |
| --- | --- | --- |
| 1 | I feel depressed, sad or hopeless. |  |
| 2 | I easily burst into tears. |  |
| 3 | I am cheerful and in good spirits. | X |
| 4 | I feel easy and carefree. | X |
| 5 | I have much less desire and enjoyment for things I usually like to do. |  |
| 6 | I have no interest in people around me. |  |
| 7 | I can laugh at funny moments. | X |
| 8 | I can enjoy pleasant things and be happy about them. | X |
| 9 | I feel exhausted and sluggish. |  |
| 10 | I can't force myself to do anything. |  |
| 11 | Decision making is easy for me. | X |
| 12 | I am full of drive and energy. | X |
| 13 | I have problems in concentrating on something. |  |
| 14 | My thoughts keep on slipping away. |  |
| 15 | I can dwell on one thing with my full concentration. | X |
| 16 | I am not easily distracted. | X |
| 17 | I am just not good enough. |  |
| 18 | Others can do things much better than I can. |  |
| 19 | I am satisfied with myself. | X |
| 20² | I take care of my appearance.^2^ | X |
| 21 | I should have done things much differently in the past. |  |
| 22 | I have made mistakes. It´s not surprising I feel bad. |  |
| 23² | I am not perfect. But who is?^2^ | X |
| 24² | I don't deserve to feel bad.^2^ | X |
| 25² | It can only get worse.^2^ |  |
| 26 | The future has nothing to offer for me. |  |
| 27 | I am looking forward to the future. | X |
| 28 | Time heals all wounds. Everything will be alright. | X |
| 29 | Sometimes I think it would be better to be dead. |  |
| 30 | I think a lot about death. |  |
| 31 | I think about putting hands on myself. |  |
| 32 | I have already thought about how to kill myself. |  |
| 33² | I sleep too much.^2^ |  |
| 34 | I have trouble falling asleep and/or wake up constantly. |  |
| 35 | My sleep was restful and sufficient. | X |
| 36 | I slept well. | X |
| 37 | I feel a constant hunger or appetite for food. |  |
| 38 | I don´t feel like eating anything. |  |
| 39 | I have a good appetite. | X |
| 40 | I eat enough and I follow a balanced diet. | X |
| 41² | I hope that I don´t get sick.^2^ |  |
| 42 | Sometimes I have an oppressive feeling in my stomach. |  |
| 43 | I am worried that something terrible will happen. |  |
| 44 | Sometimes I start panicking suddenly. |  |
| 45 | When I´m worried, I still can keep my control. | X |
| 46 | Disturbing thoughts run through my mind. |  |
| 47 | I´m calm. | X |
| 48 | When I think of my current affairs, I get anxious. |  |
| 49 | I feel safe and secure | X |
| 50 | I´m worried about something going wrong soon. |  |
| 51 | Sometimes I feel tightness in my chest. |  |
| 52 | Sometimes I can´t breathe properly. |  |

1 to be scaled from 0 = don’t agree to 5 = fully agree

2 items that should be excluded and/or reformulated
