# Supplementary material for: Validity and Reliability of the Self-administered Psycho-TherApy-SystemS (SELFPASS) Item Pool for the Daily Mood Tracking of Depressive Symptoms: Cross-sectional Web-Based Survey
Source: JMIR Ment Health. 2021 Oct 18;8(10):e29615. doi: 10.2196/29615 (PMC8561414; doi:10.2196/29615)
Supplement: Multimedia Appendix 3 [file mental_v8i10e29615_app3.docx]

| **Scale** | | **Mean**  **(N = 284)** | **SD**  **(N = 284)** | | **Item-scale-correlation** | | **Missing values**  **N (%)** | | **Number of significant inter-item-correlations (N)**  **(N = 284)** | |  |
| --- | --- | --- | --- | --- | --- | --- | --- | --- | --- | --- | --- |
| **SP-D** | |  |  | |  | |  | |  | |  |
| **No** |  |  | |  | |  | |  | |  | |
| 1 | I feel depressed, sad or hopeless. | 1.62 | | 1.45 | | 0.73^a^ | | 0 (0) | | 2 | |
| 2 | I easily burst into tears. | 1.98 | | 1.74 | | 0.35^a^ | | 0 (0) | | 8 | |
| 3 | I am cheerful and in good spirits. | 1.87 | | 1.36 | | 0.77^a^ | | 0 (0) | | 2 | |
| 4 | I feel easy and carefree. | 2.69 | | 1.46 | | 0.77^a^ | | 1 (0.4) | | 1 | |
| 5 | I have much less desire and enjoyment for things I usually like to do. | 1.65 | | 1.57 | | 0.75^a^ | | 0 (0) | | 3 | |
| 6 | I have no interest in people around me. | 1.10 | | 1.27 | | 0.40^a^ | | 0 (0) | | 13 | |
| 7 | I can laugh at funny moments. | 0.65 | | 0.97 | | 0.50^a^ | | 0 (0) | | 5 | |
| 8 | I can enjoy pleasant things and be happy about them. | 1.09 | | 1.18 | | 0.63^a^ | | 0 (0) | | 5 | |
| 9 | I feel exhausted and sluggish. | 2.16 | | 1.61 | | 0.77^a^ | | 0 (0) | | 3 | |
| 10 | I can't force myself to do anything. | 1.50 | | 1.38 | | 0.60^a^ | | 0 (0) | | 5 | |
| 11 | Decision making is easy for me. | 2.61 | | 1.51 | | 0.43^a^ | | 1 (0.4) | | 6 | |
| 12 | I am full of drive and energy. | 2.44 | | 1.46 | | 0.79^a^ | | 0 (0) | | 1 | |
| 13 | I have problems in concentrating on something. | 2.23 | | 1.63 | | 0.72^a^ | | 0 (0) | | 5 | |
| 14 | My thoughts keep on slipping away. | 2.24 | | 1.51 | | 0.65^a^ | | 1 (0.4) | | 8 | |
| 15 | I can dwell on one thing with my full concentration. | 2.07 | | 1.45 | | 0.68^a^ | | 0 (0) | | 3 | |
| 16 | I am not easily distracted. | 2.67 | | 1.47 | | 0.49^a^ | | 0 (0) | | 9 | |
| 17 | I am just not good enough. | 1.38 | | 1.49 | | 0.68^a^ | | 3 (1.1) | | 3 | |
| 18 | Others can do things much better than I can. | 2.37 | | 1.54 | | 0.49^a^ | | 1 (0.4) | | 10 | |
| 19 | I am satisfied with myself. | 2.00 | | 1.49 | | 0.75^a^ | | 1 (0.4) | | 2 | |
| 20 | I take care of my appearance. | 1.32 | | 1.08 | | **0.29^a^** | | 0 (0) | | **28** | |
| 21 | I should have done things much differently in the past. | 2.07 | | 1.67 | | 0.57^a^ | | 1 (0.4) | | 2 | |
| 22 | I have made mistakes. It´s not surprising I feel bad. | 1.27 | | 1.50 | | 0.59^a^ | | 3 (1.1) | | 2 | |
| 23 | I am not perfect. But who is? | 0.93 | | 1.06 | | **0.30^a^** | | 5 (1.8) | | **23** | |
| 24 | I don't deserve to feel bad. | 1.74 | | 1.70 | | **-0.04^a^** | | **23 (8.1)** | | 5 | |
| 25 | It can only get worse. | 1.05 | | 1.27 | | **0.30^a^** | | 7 (2.5) | | **24** | |
| 26 | The future has nothing to offer for me. | 0.83 | | 1.21 | | 0.65^a^ | | 3 (1.1) | | 1 | |
| 27 | I am looking forward to the future. | 1.46 | | 1.41 | | 0.71^a^ | | 2 (0.7) | | 3 | |
| 28 | Time heals all wounds. Everything will be alright. | 1.71 | | 1.38 | | 0.53^a^ | | 5 (1.8) | | 7 | |
| 29 | Sometimes I think it would be better to be dead. | 0.58 | | 1.21 | | 0.51^a^ | | 0 (0) | | 7 | |
| 30 | I think a lot about death. | 1.14 | | 1.42 | | 0.44^a^ | | 0 (0) | | 6 | |
| 31 | I think about putting hands on myself. | 0.29 | | 0.76 | | 0.33^a^ | | 0 (0) | | 15 | |
| 32 | I have already thought about how to kill myself. | 0.36 | | 0.95 | | 0.31^a^ | | 1 (0.4) | | 16 | |
| 33 | I sleep too much. | 1.75 | | 1.62 | | **0.20^a^** | | 1 (0.4) | | 16 | |
| 34 | I have trouble falling asleep and/or wake up constantly. | 2.05 | | 1.71 | | 0.46^a^ | | 0 (0) | | 6 | |
| 35 | My sleep was restful and sufficient. | 2.30 | | 1.52 | | 0.65^a^ | | 0 (0) | | 6 | |
| 36 | I slept well. | 2.05 | | 1.51 | | 0.59^a^ | | 0 (0) | | 6 | |
| 37 | I feel a constant hunger or appetite for food. | 2.19 | | 1.56 | | **0.09^a^** | | 0 (0) | | 4 | |
| 38 | I don´t feel like eating anything. | 0.80 | | 1.16 | | 0.38^a^ | | 0 (0) | | 8 | |
| 39 | I have a good appetite. | 1.16 | | 1.28 | | **0.22^a^** | | 1 (0.4) | | **20** | |
| 40 | I eat enough and I follow a balanced diet. | 1.33 | | 1.29 | | 0.40^a^ | | 0 (0) | | 10 | |
| **SP-A** | |  | |  | |  | |  | |  | |
| 41 | I hope that I don´t get sick. | 3.63 | | 1.51 | | **0.07^b^** | | 3 (1.1) | | 3 | |
| 42 | Sometimes I have an oppressive feeling in my stomach. | 2.00 | | 1.69 | | 0.67^b^ | | 0 (0) | | 5 | |
| 43 | I am worried that something terrible will happen. | 1.44 | | 1.49 | | 0.59^b^ | | 0 (0) | | 5 | |
| 44 | Sometimes I start panicking suddenly. | 1.45 | | 1.63 | | 0.64^b^ | | 0 (0) | | 4 | |
| 45 | When I´m worried, I still can keep my control. | 1.84 | | 1.32 | | 0.45^b^ | | 1 (0.4) | | 5 | |
| 46 | Disturbing thoughts run through my mind. | 2.00 | | 1.59 | | 0.66^b^ | | 0 (0) | | 5 | |
| 47 | I´m calm. | 2.15 | | 1.41 | | 0.64^b^ | | 0 (0) | | 2 | |
| 48 | When I think of my current affairs, I get anxious. | 2.30 | | 1.60 | | 0.64^b^ | | 1 (0.4) | | 5 | |
| 49 | I feel safe and secure | 1.85 | | 1.46 | | 0.63^b^ | | 0 (0) | | 3 | |
| 50 | I´m worried about something going wrong soon. | 1.23 | | 1.36 | | 0.58^b^ | | 1 (0.4) | | 4 | |
| 51 | Sometimes I feel tightness in my chest. | 1.77 | | 1.74 | | 0.70^b^ | | 1 (0.4) | | 4 | |
| 52 | Sometimes I can´t breathe properly. | 1.17 | | 1.51 | | 0.60^b^ | | 2 (0.7) | | 7 | |

a: N = 240; b: N=275
